# A Herpesvirus Encoded Deubiquitinase Is a Novel Neuroinvasive Determinant

**DOI:** 10.1371/journal.ppat.1000387

**Published:** 2009-04-17

**Authors:** Joy I. Lee, Patricia J. Sollars, Scott B. Baver, Gary E. Pickard, Mindy Leelawong, Gregory A. Smith

**Affiliations:** 1 Department of Microbiology-Immunology, Feinberg School of Medicine, Northwestern University, Chicago, Illinois, United States of America; 2 Department of Biomedical Sciences, Colorado State University, Fort Collins, Colorado, United States of America; Louisiana State University Health Sciences Center, United States of America

## Abstract

The neuroinvasive property of several alpha-herpesviruses underlies an uncommon infectious process that includes the establishment of life-long latent infections in sensory neurons of the peripheral nervous system. Several herpesvirus proteins are required for replication and dissemination within the nervous system, indicating that exploiting the nervous system as a niche for productive infection requires a specialized set of functions encoded by the virus. Whether initial entry into the nervous system from peripheral tissues also requires specialized viral functions is not known. Here we show that a conserved deubiquitinase domain embedded within a pseudorabies virus structural protein, pUL36, is essential for initial neural invasion, but is subsequently dispensable for transmission within and between neurons of the mammalian nervous system. These findings indicate that the deubiquitinase contributes to neurovirulence by participating in a previously unrecognized initial step in neuroinvasion.

## Author Summary

Subsets of herpesviruses, such as herpes simplex virus and pseudorabies virus, are neuroinvasive pathogens. Upon infection, these viruses efficiently target peripheral nervous system tissue and establish a life-long infection for which there is no cure. Very few pathogens are known that invade the nervous system proficiently, and the mechanism by which herpesviruses achieve neuroinvasion is largely unknown. In this study, we demonstrate that a viral protease plays a critical and specific role allowing the virus to cross the threshold of the nervous system, but is dispensable for subsequent replication and encephalitic spread within the brain.

## Introduction

Neuroinvasive herpesviruses comprise a group of pathogens with individual members infecting different mammalian hosts. Four viruses of this group infect humans: herpes simplex virus type 1 (HSV-1), herpes simplex virus type 2 (HSV-2), varicella zoster virus (VZV) and simian herpes B virus (SHBV). The latter is associated with rare life-threatening zoonotic infections. Diseases associated with these viruses range from minor recurrent lesions to shingles, keratitis and encephalitis. All of these pathologies can occur as a consequence of viral dissemination into the nervous system. There are additional neuroinvasive herpesviruses that infect mammals other than humans. Among these, pseudorabies virus (PRV) has a broad host range and is commonly associated with severe encephalitic infections, making it a useful model for studies of neuroinvasion and pathogenesis [reviewed in 1]. In addition, the propensity of PRV to transmit neuron-to-neuron during encephalitic spread has led to its use as a self-amplifying tracer for mapping of the vertebrate neural circuitry [reviewed in 2]. PRV is closely related to VZV (both are members of the varicellovirus subgroup of the alpha-herpesvirus family), and shares a common structure, similar genetic composition and a related infectious cycle with all neuroinvasive herpesviruses [2],[3].

To date, mutant viruses displaying defects in neurotropism fall into two categories: neuron-specific replication mutants and axon transport mutants. Mutants of HSV-1 that fail to replicate in neurons include viruses lacking thymidine kinase activity or ICP34.5 [4]–[7]. In contrast, HSV or PRV lacking the gE glycoprotein or the Us9 type-II membrane protein replicate in neurons, but subsequently fail to transport progeny viral particles within axons [8]–[12].

Recently, an isolate of PRV mutated in a conserved deubiquitinase (DUB) domain of a viral structural protein, the pUL36 tegument protein, was reported to infect the nervous system with delayed kinetics following intranasal inoculation into mice [13]. In this report, we demonstrate that an isolate of PRV mutated in a critical catalytic residue of the pUL36 DUB domain replicates and spreads within the nervous system, but is severely attenuated in its ability to initially invade the nervous system from peripheral tissues. Using time-lapse fluorescence microscopy, the DUB mutant virus was found to transport in axons both following entry (retrograde transport) and replication (anterograde transport) in cultured neurons. Furthermore, the DUB mutant replicated and spread in the nervous system following direct inoculation: either by injecting mutant virus into the brain or vitreous chamber of the eye. Unlike previously described neuroinvasive mutant viruses, the DUB mutant was competent to spread to synaptically-linked second- and third-order CNS neurons in a manner similar to wild-type infections in both anterograde and retrograde circuitry. Together, these findings demonstrate that the pUL36 deubiquitinase represents a novel neuroinvasion determinant that functions at a previously unrecognized initial step in penetration of the nervous system from peripheral tissues.

## Results

### Isolation and initial characterization of the pUL36 mutant virus

**Figure 1:**
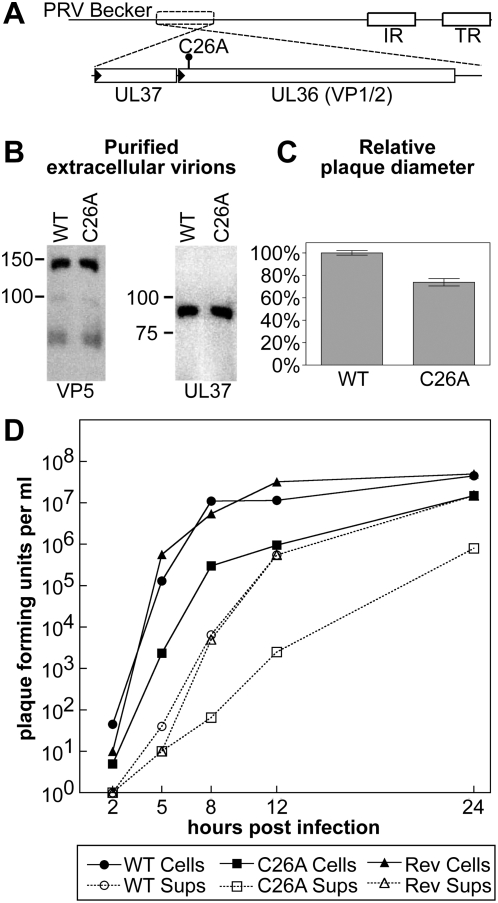
Propagation of PRV with a mutated amino terminus in cultured cells. (A) Illustration of the PRV-Becker genome with region encoding the UL36 gene (which encodes the pUL36 protein) and neighboring UL37 gene expanded. The position of the codon change resulting in the C26A point mutation is indicated. Promoters are represented as black triangles. IR, internal repeat. TR, terminal repeat. (B) Purified extracellular virions encoding mRFP1-capsids and either a wild-type UL36 allele (WT; PRV-GS847) or UL36 allele encoding the amino-terminal point mutation (C26A; PRV-GS1652) were examined by Western blot analysis for the incorporation of the UL37 protein. The major capsid protein, VP5, was used as a loading control. (C) Comparison of plaque diameters resulting from infection of Vero cells with the virus encoding wild-type UL36 (WT) or mutated UL36 (C26A) allele. Each virus also encodes the mRFP1-VP26 fusion (red capsids). Error bars are standard error of the means (SEM). (D) Propagation kinetics of viruses encoding wild-type UL36 (WT), mutated UL36 (C26A), or a wild-type revertant of the C26A allele (Rev). All viruses also encode the mRFP1-VP26 fusion (red capsids). Infectious virions were detected as plaque-forming units harvested from either the cells (cells) or tissue culture supernatant (sups).

A conserved cysteine residue in the pUL36 tegument protein that is critical for the proteolytic activity responsible for deubiquitination [14]–[18] is also required for efficient invasion of the central nervous system by PRV [13]. To examine the neuroinvasive defect further, an alanine substitution (C26A) was incorporated into an isolate of PRV that also encodes a red-fluorescent protein fused to the VP26 capsid protein (Figure 1A). The mRFP1-VP26 fusion allows for imaging of viral particles in neurons and does not impact viral propagation in cell culture [19],[20]. The C26A mutation did not have a polar effect on the upstream expression of the UL37 gene (Figure 1B), unlike a previously described in-frame deletion that removes the amino-terminal DUB domain from the pUL36 protein [21]. The kinetics of propagation and cell-to-cell spread of PRV encoding the DUB C26A mutation were reduced relative to the virus encoding wild-type pUL36, consistent with previous findings that the pUL36 DUB serves an important, but not essential, function in cell culture (Figure 1C and 1D) [16], [21]–[23].

### Neuroinvasion in an eye model of infection

PRV is frequently used as a trans-synaptic neurotracer to map circuits within the mammalian nervous system [reviewed in 2]. In particular, neural circuits between the eye and brain have been extensively studied in rodents infected with PRV, which we used as the basis for our current studies of the C26A mutant virus [24]–[33]. Viruses encoding RFP-capsids and either a wild-type pUL36 allele (PRV-GS847) or the C26A allele of pUL36 (PRV-GS1652) were injected into the vitreous body of the eye of adult rats to directly expose retinal ganglion cell (RGC) neurons to the inoculum. RGCs project axons to visual centers in the brain and provide an anterograde route for encephalitic spread of the virus. Of the nine animals infected with wild-type virus, 4 succumbed to infection between 60-74 hours post infection (hpi); the remaining 5 animals were killed between 70–72 hpi. The C26A virus was attenuated in this model with animals showing little ill effect; therefore, animals were killed 47–264 hpi to examine the extent of viral spread. Because several attenuated mutants of PRV fail to traverse anterograde circuits, the brains of infected animals were examined for capsid fluorescence [10],[24],[28]. The superior colliculus (SC), a primary target of RGC efferent axons, contained trans-synaptically labeled neurons in animals infected with either the wild-type or mutant virus in about 25% of the intravitreally injected animals (Table 1), which is similar to other wild-type strains of PRV [24].

**Table 1:** Summary of PRV neuroinvasion by examination of trans-synaptically labeled neurons.

	WT	C26A	Rev (C26)	1∶1^d^	PRV-152
Anterograde (SC)^a^					
Intravitreal^b^	1/5 (20%)	4/15 (27%)	n/d	n/d	n/d
Retrograde (EW)^a^					
Intravitreal^b^	5/5 (100%)	0/15 (0%)^c^	n/d	n/d	n/d
Anterior Chamber^b^	3/3 (100%)	0/4 (0%)	3/3 (100%)	3/3 (100%)^e^	4/4 (100%)
Retrograde (RGC)^a^					
SCN^b^	n/d	2/2 (100%)	n/d	n/d	n/d
Retrograde (CNS)^a^					
Eyelid^b^	n/d	1/11 (9%)	n/d	n/d	10/11 (91%)

a site imaged.

b site injected.

c one animal showed fluorescence signal in the oculomotor nucleus.

d 1∶1 mixture of PRV-152 (Bartha; green fluorescence) and PRV-GS1652 (C26A; red fluorescence).

e all 3 animals emitted red and green fluorescence in the EW indicative of PRV-152 and PRV-GS1652 co-infection.

SC superior colliculus.

EW Edinger-Westphal nucleus.

RGC retinal ganglion cells.

CNS central nervous system.

WT virus encoding wild-type pUL36.

C26A virus encoding C26A mutant isoform of pUL36.

Rev revertant of C26A virus (encodes wild-type pUL36).

n/d no data.

Because anterograde spread of the C26A mutant of PRV was unexpected, and did not account for its severe attenuation, the brains of these animals were also examined for evidence of infection via retrograde autonomic circuits. Intravitreal injection into the eye can expose the iris and ciliary body to viral inoculum, which receive autonomic innervation. The time course of infection and retrograde spread of PRV in these circuits is somewhat variable due to the gelatinous matrix of the vitreous body (compared to the aqueous humor of the anterior chamber, see below). Nevertheless, in these experiments wild-type virus was reproducibly observed to have infected the Edinger-Westphal nucleus (EW) of the midbrain, preganglionic parasympathetic neurons that project to neurons in the ciliary ganglion via the oculomotor nerve, which in turn innervates the smooth muscles in the iris and ciliary body in the eye to mediate pupillary constriction and lens accommodation, respectively. In contrast, capsid fluorescence was never detected in the EW following C26A virus infection (Table 1). Wild-type virus also spread to the paraventricular nucleus (PVN) via a retrograde circuit of sympathetic origin (from iris, to superior cervical ganglion to spinal cord to paraventricular nucleus). Consistent with an inability to spread in retrograde circuitry, no labeled cells were observed in the PVN of C26A virus infected animals (data not shown). However, one animal that received an intravitreal injection of C26A virus showed clear signs of retrograde spread to the oculomotor nucleus, which innervates 4 of the 6 extraocular muscles of the eye (data not shown). While this result was only observed once, it cannot be ignored as it indicates that the C26A virus was capable of spreading from the eye to the brain via a retrograde circuit under some circumstances. We hypothesized that damage to the sclera (outer layer of the eye) and attached extraocular muscles resulting from intravitreal injection may sometimes allow for spread from the extraocular muscle to the oculomotor nucleus in the brain. Therefore, further examination of retrograde spread was necessary to determine the specific defect resulting from the C26A mutation.

### The C26A virus is competent to spread in anterograde and retrograde circuits

**Figure 2:**
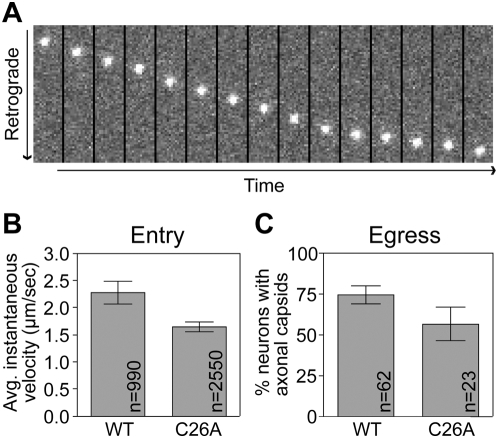
Viral transport dynamics in axons of cultured primary neurons. (A) Representative example of retrograde transport of an individual capsid in a dorsal root sensory axon after infection with the C26A virus, shown as a time-lapse montage. All frames are 1.68×10.8 µm. (B) Retrograde transport efficiency measured as frame-by-frame velocities of individual capsid particles in axons (as documented in panel A). (C) Anterograde transport efficiency measured by accumulation of newly replicated capsids in axons.

To determine if the C26A virus was capable of intracellular transport in both the retrograde and anterograde directions, we examined axonal transport in cultured dorsal root sensory neurons by tracking red-fluorescence emissions from capsid particles by time-lapse microscopy. In these assays, the C26A virus behaved similarly to the wild-type virus, having only small decreases in axon transport during both stages of infection (Figure 2). These findings demonstrate that intracellular transport of the C26A virus was intact, and suggests the defect observed after intravitreal injection was likely due to a change in intercellular viral spread in animals. While these results did not immediately explain the defect observed in spread through retrograde sympathetic and parasympathetic circuits following intravitreal inoculation (i.e. to the EW and PVN), they were consistent with the one instance of C26A virus spread to the oculomotor region of the midbrain.

**Figure 3:**
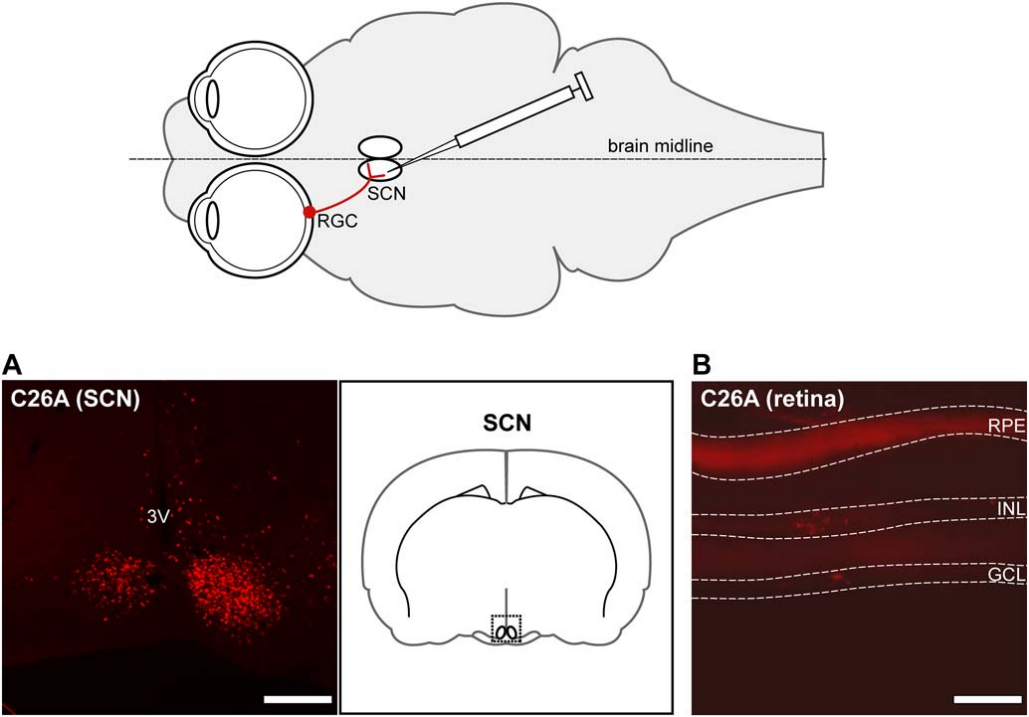
Retrograde transmission following CNS injection. Stereotactic injection route resulting in exposure of viral inoculum to SCN neurons. RGC ipsilateral projection to the SCN indicates route of viral transmission to the eye. (A) Representative image of virus fluorescence in the SCN of a coronal brain slice (region imaged is indicated by the doted box in right panel). 3 V, third ventricle. Scale bar = 40 µm. (B) Virus detected in the eye following retrograde transmission from the SCN is seen as punctate fluorescence in the RGCs of the ganglion cell layer (GCL) and in bipolar/amacrine cells of the inner nuclear layer (INL) of the retina. The bright fluorescent band near the top of the image is autofluorescence emitted from the retinal pigmented epithelium (RPE) at the back of the retina, and is not of viral origin. Scale bar = 10 µm.

To further examine trans-synaptic retrograde transport *in vivo*, stereotaxic injection of virus directly into the brain was employed. The suprachiasmatic nucleus (SCN), which receives axon projections from RGCs in the eye [34], was injected with the C26A virus. Although there is one SCN in each hemisphere of the brain, and both neighbor each other adjacent to the brain's midline, the stereotactic injection procedure allowed for delivery of virus to the SCN unilaterally. C26A virus infection was prominent in the injected SCN, and to a lesser extent in the contralateral SCN. This spread is noteworthy, as the SCN are reciprocally innervated [34], and viral spread to the neighboring SCN provided further evidence that the C26A virus was competent to spread trans-synaptically in the CNS. Examining the retinas of the SCN-injected animals provided a definitive confirmation of retrograde transneuronal transmission with the C26A virus: capsid fluorescence indicative of infected neurons was detected in neurons in the RGC layer and also in bipolar and/or amacrine cells (neurons presynaptic to RGCs) located in the inner nuclear layer of the retina (Figure 3). This finding was consistent with the studies using cultured neurons, and unambiguously demonstrated that C26A virus is capable of multiple rounds of retrograde trans-synaptic spread *in vivo* (from SCN neurons in the brain to RGCs in the eye to bipolar/amacrine cells in the eye).

### The C26A defect occurs after virus reaches and replicates in peripheral sites of innervation

**Figure 4:**
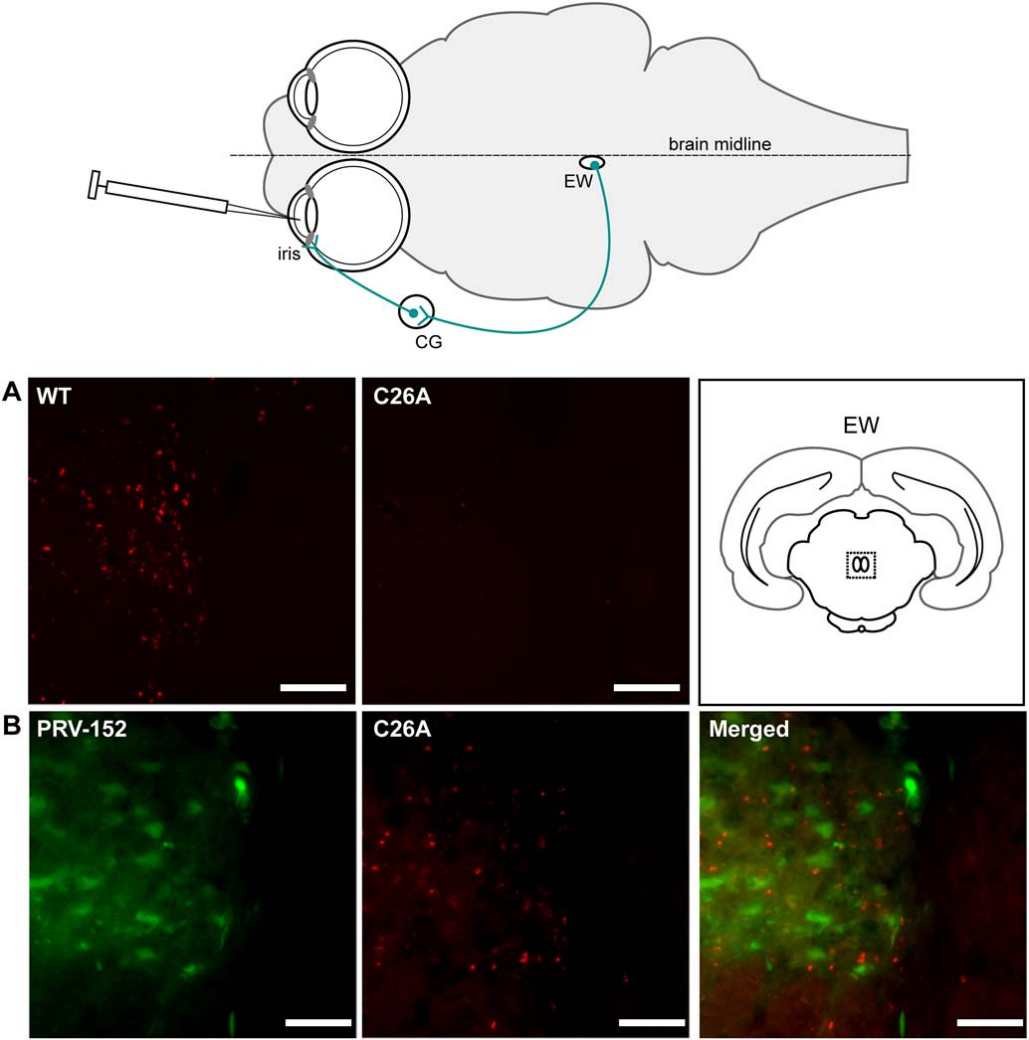
Retrograde transmission defect following anterior chamber injection. Anterior chamber injection route resulting in exposure of viral inoculum to the iris. The route of viral encephalitic spread is indicated: autonomic oculomotor nerve innervation of the iris from the ciliary ganglion (CG), which in turn receives innervation from parasympathetic neurons of the Edinger-Westphal nucleus (EW) of the midbrain. (A) Representative coronal images of EW (shown as a dashed box in coronal illustration) following anterior chamber injection or either wild-type or C26A virus. (B) Co-infection with PRV-152 and the C26A virus. Diffused GFP fluorescence and punctate RFP capsid signals are emitted from PRV-152 and the C26A viruses, respectively. Scale bars = 10 µm.

The data up to this point indicated that the C26A virus was incapable of spread in a subset of neural circuits, but the basis for the selective loss of function could not strictly be attributed to an inability to transport in either retrograde or anterograde circuitry. Because the C26A defect was observed only in retrograde circuits innervating tissues in the anterior chamber of the eye (i.e. iris and ciliary body), which are less efficiently infected following intravitreal injections, animals were next injected in the anterior chamber of the eye directly. This infection route immediately exposes the iris and neighboring ciliary body to the viral inoculum and provides a more reliable infection of the EW with all neuroinvasive strains of PRV examined to date [25]. Consistent with the intravitreal injections, the C26A virus again failed to spread to the brain; no infection was noted in the EW or any other area of the brain in all instances (Figure 4A). In contrast, a revertant of the C26A virus was unimpaired for spread to the EW (Table 1).

We next considered that the C26A virus typically propagated to titers 10-fold reduced relative to wild type (Figure 1). Preliminary studies indicated that the decrease in titer correlated to an increase in the particle∶PFU ratio of the C26A virus, as the C26A virus produced approximately equal numbers of fluorescent viral particles as the wild-type virus (data not shown). Therefore, by concentrating the C26A stocks to attain titers comparable to the wild-type virus, which were necessary to initiate reproducible infections of the nervous system, defective particles in the concentrated C26A viral stock may have interfered with infection of the retrograde circuits. As an initial test for interference, a stock of wild-type virus (PRV-GS847) was prepared as a concentrated stock and inoculated into the anterior chamber of the eye. Although 10-fold more viral particles were inoculated in this experiment than normal, retrograde transmission to the EW occurred unabated (data not shown). As a second test for interference, the C26A virus was mixed with an equal titer of PRV-152 virus and co-injected into the anterior chamber of the eye. PRV-152 is a derivative of the PRV-Bartha strain, which is an attenuated PRV isolate that is incapable of spread in anterograde circuits but retains spread in all retrograde circuitry [24]. PRV-152 encodes a GFP reporter cassette driven by a cytomegalovirus immediate early promoter, and therefore provides a fluorescent marker that is distinct both spectrally and spatially from the RFP-capsid emissions of the C26A virus (mRFP1-capsid fluorescence is most profound in the nucleus of an infected cell, whereas the GFP signal is diffuse throughout the cell body and neurites of an infected neuron). If the concentrated stock of C26A virus inhibited infection of the sympathetic and parasympathetic circuits innervating the eye, PRV-152 infection of the same circuits should also be blocked in a co-infection paradigm. However, interference was never observed and GFP fluorescence indicating presence of PRV-152 was readily detected in the EW (Figure 4B). Furthermore, RFP-capsid fluorescence emissions indicative of the C26A virus were now also detected in the EW of these co-injected animals.

**Figure 5:**
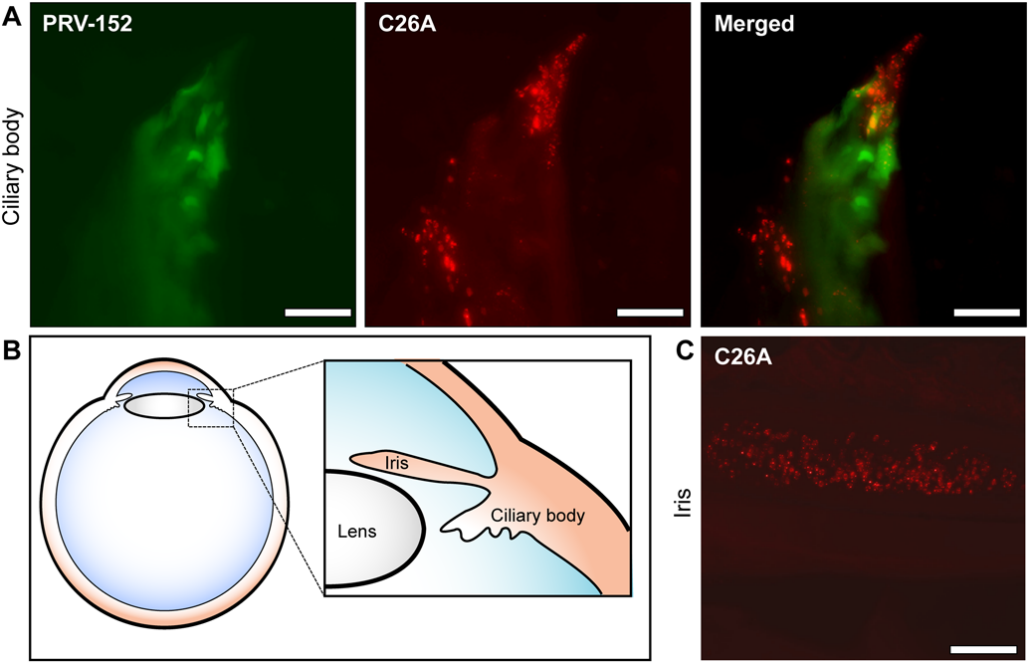
Viral infection in peripheral tissue of the eye. (A) Images of the ciliary body following co-infection with PRV-152 (diffuse GFP signal) and the C26A virus (punctate RFP signal) in the anterior chamber. Cells infected with both viruses are evident in the merged image. (B) Illustration of the peripheral tissues in the eye (iris and ciliary body) exposed to viral inoculum and imaged in these studies. (C) C26A virus fluorescence from nuclei of cells in the iris following anterior chamber injection. Scale bars = 10 µm.

Spread of C26A virus to the EW in the co-injection experiment indicated that PRV-152 had complemented the C26A defect. For complementation to occur, both viruses would have had to replicate in a common cell prior to entering the nervous system. This provided an incentive to examine the iris and ciliary body of the co-injected animals for co-infected cells (Figure 5A and 5B). Infection of the ciliary body and iris by both PRV-152 and the C26A virus was frequently observed and areas infected by either virus often displayed some degree of overlap, indicating that initial replication in these tissues prior to neuroinvasion provided an opportunity for the C26A virus to benefit from the unaltered pUL36 protein expressed by PRV-152 (Figure 5A, and data not shown). In a subsequent experiment, the C26A virus was found to infect cells of the iris efficiently when injected alone into the anterior chamber of the eye (Figure 5C). Therefore, the C26A virus remained competent to enter and replicate in the iris, but in the absence of PRV-152 failed to invade the nervous system via the autonomic innervation of the iris/ciliary body.

### The C26A virus is defective for neuroinvasion from peripheral tissues

**Figure 6:**
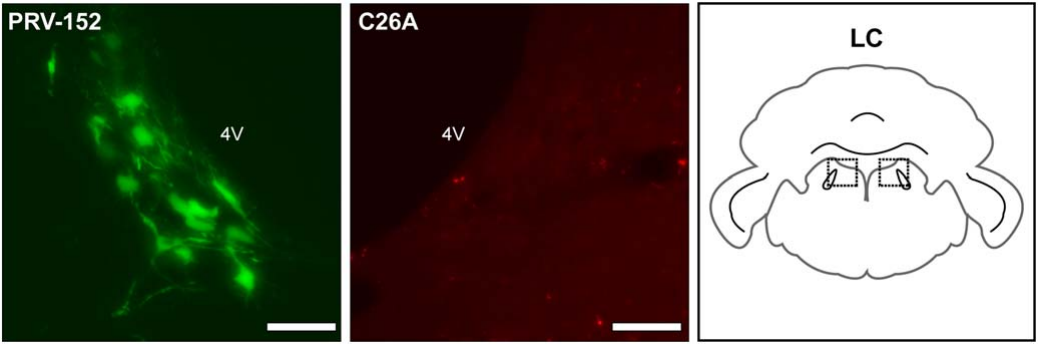
Retrograde transmission defect following eyelid injection. Example of a single animal injected with PRV-152 and the C26A virus independently injected in opposing eyelids. Retrograde transmission of PRV-152, but not the C26A virus, to the locus cerulus (LC) is indicated in the fluorescence images. Region of brain imaged is illustrated as the two dashed boxes in a coronal brain slice (right panel). 4 V, fourth ventricle. Scale bar = 10 µm.

To confirm that the C26A virus had a specific defect in neuroinvasion from tissues innervated by the PNS, PRV-152 and the C26A virus were independently injected into the skin and muscle of one eyelid each per animal; the smooth muscle of the eyelid receives both sympathetic and parasympathetic innervation [35]. In this infection paradigm, PRV-152 consistently spread to several regions of the brain. Most notable was transmission to the locus ceruleus of the caudal pons (Figure 6). The C26A virus failed to spread to the locus ceruleus, and in fact was entirely absent from the brain in 10 of 11 animals (Table 1).

To explain how the C26A virus spread retrogradely in one animal, we again considered the possibility that injection into the eyelid might have caused local tissue damage that allowed direct exposure of the inoculum to innervating axon projections. We expect that a similar event likely occurred in the one intravitreal injected animal in which C26A virus spread specifically to the oculomotor nucleus (see above). To determine if injection can, in some instances, allow for direct exposure of the inoculum to axons embedded within the peripheral tissue, the beta subunit of cholera toxin (CTB) conjugated to Alexa Fluor 594 was injected into the eyelid. CTB is a retrograde tracer, but unlike neuroinvasive herpesviruses which spread trans-synaptically, CTB labeling is restricted to the neuron cell body from which an axon projects. Furthermore, CTB must come into direct contact with the axon to label the distal neuronal cell body. Of 6 animals tested, only one showed CTB retrograde label into the brain. The absence of CNS labeling in the remaining five animals indicated that injection into the eyelid infrequently allowed for direct exposure to nerve endings. This finding was consistent with the conclusion that the C26A virus is specifically incapable of invading the mammalian nervous system from tissues innervated by the PNS, except in infrequent cases when nerve endings become directly exposed to the inoculum.

## Discussion

Since its discovery four years ago [14], the herpesvirus pUL36 deubiquitinase has been the focus of several reports due to its potential as a target for the development of novel antiviral compounds. The DUB is conserved in all herpesviruses so far examined, and enzymatic assays are available to aid in screens for small molecule inhibitors [15],[36]. However, little is known regarding the role of the DUB in the herpesvirus infectious cycle and its significance to productive infection, and substrates of the DUB during infection have yet to be identified. Surprisingly, the DUB is one of the few domains of the large pUL36 tegument protein (an essential herpesvirus structural component) that is dispensable for herpesvirus propagation in cultured cells. Although mutation of the DUB in several herpesviruses reduces viral propagation kinetics, the underlying defect associated with these mutants in cultured cells is unclear [16], [21]–[23]. Initial studies of DUB-mutant herpesviruses in animal models have revealed more substantial defects. MDV, a herpesvirus noted for its oncogenic activity, requires the DUB to produce tumors in chickens [23]. The neuroinvasive herpesvirus studied in this report, PRV, requires the DUB to efficiently invade the nervous system following intranasal inoculation in mice [13]. Because the underlying deficiencies that produce these fundamental losses in pathogenesis are not understood, we sought to explore the PRV neuroinvasive defect in greater detail. Although the DUB activity in PRV has yet to be studied enzymatically, the conservation of the DUB activity throughout all three branches of the herpesviridae indicates that the DUB is important for the pathogenesis of these viruses [14]–[17],[23].

Our initial experiments produced results consistent with a selective loss of retrograde trafficking in the nervous system; however, retrograde transport of the C26A virus was unmasked by either infecting neurons directly in the retina or brain, or presumably by mechanical damage at the site of injection. An important insight was fortuitously made when the C26A mutant was co-infected with a virus encoding a wild-type pUL36 protein (PRV-Bartha), resulting in the rescue of the C26A defect and retrograde spread to the brain. This trans-complementation could only occur if the C26A virus remained competent to infect cells prior to neuroinvasion, which was subsequently confirmed by examining the eye for infection. Therefore, the C26A mutation resulted in a virus that replicated in non-neuronal cells both in culture and in the iris, and also replicated and spread in cultured neurons and neural circuits *in vivo*, but largely failed to spread between the innervated non-neuronal tissue and the innervating neurons. Such a defect has never been observed for a neuroinvasive herpesvirus before, and reveals that a previously unrecognized barrier to viral dissemination into the nervous system is overcome by the amino-terminal deubiquitinase domain embedded in the pUL36 protein. The neuroinvasion defect was rescued by either co-infection with a virus encoding a wild-type pUL36 protein or by repairing the C26A mutation directly, confirming that the neuroinvasive defect mapped to the pUL36-encoded DUB.

In contrast to the results presented here, a previous study that examined the role of the PRV DUB following intranasal infection reported only a general delay in neuroinvasion [13]. This apparent discrepancy can be explained by findings made with a replication-defective mutant of herpes simplex virus type 2 (HSV-2), a neuroinvasive herpesvirus related to PRV. Although a replication-defective virus cannot propagate and spread infection cell to cell, the HSV-2 replication-defective mutant nevertheless infected the nervous system following intranasal inoculation in mice, although not as efficiently as wild-type HSV-2 [37]. Because the same HSV-2 mutant was not detected in the nervous system following intradermal or intramuscular inoculation, these findings indicate that neurons are directly exposed to viral inoculum in the nasal cavity. Therefore, neuroinvasive defects can be partially obscured by a low level of direct neuron exposure in intranasal infection models, which likely explains the previous PRV findings [13]. Direct exposure of neurons was also occasionally a complication in our eye and eyelid injection paradigms but in these instances was presumably a result of tissue damage from the injection procedure, which is consistent with cholera toxin sporadically labeling neurons following injection of some animals.

Identification of the molecular determinants that promote the neuroinvasive behavior of this class of herpesviruses not only provides insight into the underlying mechanisms of viral dissemination within host tissues, but also into the relationship between tissue tropism and pathogenesis. Yet, the majority of herpesviruses are not neuroinvasive pathogens, which argues that the pUL36 DUB may perform a more fundamental role during infection. Infection of cultured cells with DUB-mutant herpesviruses has revealed delays in viral assembly, propagation kinetics and cell-cell spread [13],[16],[23]; however, how these generalized defects relate to the specific phenotype seen in animals reported here is unclear. One possible role for the DUB is cell-to-cell transmission of infection. All herpesviruses are noted for their ability to establish latent infections in cell types other than those first exposed to the virus during natural infections. For example, Epstein-Barr virus first infects epithelial cells in lymphoepithelial structures such as the tonsils, and then spreads to B-lymphocytes where latency is established. The general propensity of herpesviruses to transfer from one cell type to another across presumably specialized regions of cell-cell contact may have an underlying requirement for the deubiquitinase activity of pUL36. Because pUL36 is a cytosolic protein, a possible mode of action would be to promote newly assembled intracellular virions to exit cells at sites of cell-cell contact that would otherwise be obscured from the virus. We expect that 2-chamber models will allow further examination of the DUB mutant in epithelia-neuron transmission in culture; however, this will require developing a system where neurites can be effectively excluded from exposure to the initial viral inoculum [8], [38]–[42].

The pUL36 tegument protein is greater than 300 kDa in neuroinvasive herpesviruses such as HSV and PRV, with the amino-terminal DUB amounting to less than 1/10^th^ of the protein's mass. In addition to pUL36 being a critical component of the herpesvirus structure that links the capsid to the tegument and envelope layers of the virion [43]–[48], pUL36 is also implicated in several stages of the infectious cycle. These stages include: delivery of viral DNA into the nucleus [49]–[51], capsid egress from nuclei [52], capsid microtubule-based transport [52], and virion assembly and egress from cells [45],[46]. Although the DUB could be fortuitously embedded in the pUL36 protein, it will be of interest to determine if there is more than just a casual link between neuroinvasion and other pUL36 functions. Understanding the mode of action of the pUL36 amino terminus, and identifying natural cellular and viral substrates for the deubiquitinase enzymatic activity will be essential to resolving how herpesviruses spread in animals and cause disease.

## Materials and Methods

### Viruses

Two recombinant viruses were made from a derivative of the PRV (Becker strain) infectious clone, pBecker3, which was previously made to encode an mRFP1-VP26 (red-fluorescent capsid) fusion allele (pGS847) [20],[53]. A mutant derivative of pGS847 encoding a single codon change in the UL36 gene (C26A) was constructed using a two-step RED recombination protocol [54]. The primers used for this protocol were 5′- TCAGTATGACCCCGACCTGGGGCCCGGGTCGGGCGTCTCG**GCT**CTGCGCTCCTCCCTCTCCTTAGGATGACGACGATAAGTAGGG and 5′- TCGTGAAGACCAGGCGCAGGAAGGAGAGGGAGGAGCGCAG**AGC**CGAGACGCCCGACCCGGGCCCAACCAATTAACCAATTCTGATTAG . The primers encode the sequence to be mutated (bold) with surrounding homology to the UL36 gene, as well as homology to the pEPkan-S template plasmid (underlined). Transfection of the resultant infectious clone, pGS1652, into pig kidney epithelial (PK15) cells produced the PRV-GS1652 virus, which typically propagated to a titer of 1×10^7^ plaque forming units (PFU) per ml. A revertant virus, PRV-GS1768, was constructed using the same methods. The primers used were 5′- TCAGTATGACCCCGACCTGGGGCCCGGGTCGGGCGTCTCG**TGC**CTGCGCTCCTCCCTCTCCTTAGGATGACGACGATAAGTAGGG and 5′- TCGTGAAGACCAGGCGCAGGAAGGAGAGGGAGGAGCGCAG**GCA**CGAGACGCCCGACCCGGGCCCAACCAATTAACCAATTCTGATTAG . All mutations were confirmed by restriction digest analysis and sequencing.

PRV152 is a derivative of the PRV-Bartha vaccine strain that encodes a GFP-expression cassette driven by the CMV immediate early promoter inserted in the gG locus, and was described previously [33]. PRV152 spreads through retrograde circuits efficiently, and served as a control for these studies.

### Preparation of viral stocks and western blot analysis

Stocks of PRV-GS847 (WT), PRV-GS1652 (C26A), PRV-GS1768 (revertant) and PRV152 (Bartha-GFP) were harvested from infected PK15 cells grown in DMEM supplemented with 2% bovine growth supplement (Invitrogen). High titer virus stocks were prepared for a subset of animal studies as described previously [21]. For Western blot analysis of viral protein incorporation into extracellular virions, virions were purified from infected cell supernatants by rate zonal ultracentrifugation in a 12–32% dextran gradient as described previously [55]. The VP5 capsid protein was detected with mouse monoclonal antibody 3C10 (a gift from Lynn Enquist) at a dilution of 1∶1000 and the UL37 tegument protein was detected with rabbit polyclonal antibody D1789 at a dilution of 1∶2500, as described previously [56].

### Viral propagation kinetics, titers, and plaque size analysis

Quantitation of viral propagation kinetics by single-step growth curve analysis was conducted as previously described, with the exception that Vero cells were substituted for PK15 cells [57]. Vero cells were infected at a multiplicity of infection (MOI) of 5 for each viral strain. Viral titers from cells or media supernatants harvested at 2, 5, 8, 12 or 24 hours post infection (hpi) were determined in duplicate by plaque assay. Plaques were counted following infection of cells in 6-well trays with serial 10-fold dilutions for each harvested sample. The diameters of these plaques were also measured from images captured with a 10× objective with a 0.3 numerical aperture (NA). Two orthogonal diameter measurements of each fluorescent plaque were analyzed using the Metamorph software package (Molecular Devices). The reported plaque diameters represent an averaged of more than 50 plaques per virus, and were plotted using the Prism software package (GraphPad Software).

### Live-cell fluorescence microscopy

Imaging of entry and egress axonal transport events was performed using primary cultured neurons from embryonic chicken (E8–E10) dorsal root ganglia, as previously described [21]. Metamorph and Prism software packages were used for the analysis of instantaneous velocities and quantitation of viral capsids in axons. Time-lapse images were captured using an inverted wide-field Nikon Eclipse TE2000-U microscope fitted with a 60×/1.4 NA objective and a Cascade:650 charge-coupled device (Roper Scientific). The microscope was housed in a 37°C environmental box (Life Imaging Services).

### Animals

Male Sprague-Dawley and Long Evans rats (Charles River Breeding Laboratories) were maintained under a light/dark cycle of 12 h light/12 h dark with food and water available *ad libitum*. Animals were at least 14 weeks of age when used in experiments.

### In vivo injection of virus

#### Intraocular injections

Under isoflurane inhalation anesthetic (2.5–5%), animals received a unilateral intravitreal injection of 2 µl of either PRV-GS847 or PRV-GS1652 (1–14.5×10^8^ plaque-forming units/ml) or a unilateral anterior chamber injection of the same quantity of PRV-GS847, PRV-GS1652, PRV152 or a 1∶1 mixture of PRV-GS1652:PRV152, over a 1-min interval using a Nanoject II nanoinjector fitted with a glass micropipette (Drummond Scientific Co, Broomall, PA). A fresh stock of virus was thawed for each injection. Animals were maintained in a biosafety level 2 facility for up to 11 days post-inoculation.

#### Intracranial injections

Under isoflurane inhalation anesthetic (2.5–5%), animals were positioned in a Kopf stereotaxic apparatus (David Kopf Instruments, Tujunga, CA). A craniotomy was performed, the dura was exposed and a zero depth reading was made prior to the dura mater being excised. A glass micropipette attached to a Nanoject II nanoinjector (Drummond Scientific Co, Broomall, PA), angled laterally 10° to the vertical to avoid the midline third ventricle, was lowered into the hypothalamus to a region slightly dorsal to the suprachiasmatic nucleus (SCN) using coordinates determined empirically in test subjects. The micropipette was lowered 7.7–7.8 mm from the dura mater, thus avoiding involvement of the retinal axons in the optic chiasm, and approximately 200 nl (the Nanoject II was set to the 207 nl volume preset) of a 1×10^8^ pfu/ml stock of PRV-GS1652 were ejected into the hypothalamic neuropil. The micropipette was left in place for about 1 minute before slowing retracting, and the craniotomy was packed with gelfoam and the incision was sutured.

#### Eyelid injections

Under isoflurane inhalation anesthetic (2.5–5%), the skin of the upper eyelid of one eye was injected with 2 µl of PRV-GS1652 (1–4.25×10^8^ pfu/ml) and the upper eyelid of the other eye was injected with 2 µl of PRV152 (1×10^8^ pfu/ml). In a separate set of experiments, animals received a unilateral upper eyelid injection of 2 µl of cholera toxin B subunit conjugated to Alexa Fluor 594 (Molecular Probes, Eugene, OR) using a Nanoject II nanoinjector fitted with a glass micropipette (Drummond Scientific Co, Broomall, PA).

### Tissue preparation

After post-injection intervals ranging from 2–11 days, animals were deeply anesthetized with sodium pentobarbital (80 mg/kg, i.p.), and perfused transcardially with 0.9% saline followed by freshly prepared fixative consisting of 4% paraformaldehyde in phosphate buffer (0.1 M), pH 7.3. Brains were removed, stored in the same fixative containing 30% sucrose at 4°C overnight, and sectioned at 40 µm in the coronal plane on a sliding microtome equipped with a freezing stage (Physitemp Instruments Inc., Clifton, NJ). Sections were collected in phosphate buffer, mounted on subbed slides, blotted to remove excess buffer, and coverslipped with Vectashield mounting medium (Vector Laboratories, Burlingame, CA). Coverslips were sealed with fingernail polish to prevent dehydration, and slides were stored in the dark at 4°C. EGFP and mRFP1 fluorescence is stable under these conditions for several months with minimal quenching. Slides were examined using a Leica (Nussloch, Germany) DMRA light microscope equipped with epifluorescence and fitted with a microstepping servomotor in the z–axis. Images were captured using a Hamamatsu (Hamamatsu City, Japan) C4742-95 CCD digital camera under epifluorescence using either EGFP optics (412020 High Q narrow band EGFP filter; Chroma, Brattleboro, VT) or mRFP optics (#41004 HQ Texas Red filter, Chroma) and deconvolved using Openlab fluorescence deconvolution software (Improvision, Boston, MA) running on an Apple Macintosh G4 platform. Digital images were pseudo-colored, and images were prepared using Adobe Photoshop version 6.0.1. Images were enhanced for brightness and contrast.
